# Imported Human Rabies Cases Worldwide, 1990–2012

**DOI:** 10.1371/journal.pntd.0002209

**Published:** 2013-05-02

**Authors:** Philippe Carrara, Phillipe Parola, Phillipe Brouqui, Philippe Gautret

**Affiliations:** 1 Assistance Publique Hôpitaux de Marseille, CHU Nord, Pôle Infectieux, Institut Hospitalo-Universitaire Méditerranée Infection, Marseille, France; 2 Aix Marseille Université, Unité de Recherche en Maladies Infectieuses et Tropicales Emergentes (URMITE), UM63, CNRS 7278, IRD 198, Inserm 1095, Faculté de Médecine, Marseille, France; The Global Alliance for Rabies Control, United States of America

## Abstract

Sixty cases of human rabies in international travelers were reviewed from 1990–2012. A significant proportion of the cases were observed in migrants or their descendants when emigrating from their country of origin or after a trip to visit friends and relatives or for other reasons (43.3%). The cases were not necessarily associated with long-term travel or expatriation to endemic countries; moreover, cases were observed in travelers after short trips of two weeks or less. A predominance of male patients was observed (75.0%). The proportion of children was low (11.7%). Cases from India and Philippines were frequent (16 cases/60). In a significant proportion of cases (51.1%), diagnosis was challenging, with multiple missed diagnoses and transfers from ward to ward before the final diagnosis of rabies. Among the 28 patients whose confirmed diagnosis was obtained ante-mortem, the mean time between hospitalization and diagnosis was 7.7 days (median time: 6.0 days, range 2–30) including four cases with a diagnosis delayed by 15 or more days. In five cases, a patient traveled through one or more countries before ultimately being hospitalized. Three factors played a role in delaying the diagnosis of rabies in a number of cases: (i) a low index of suspicion for rabies in countries where the disease has been eradicated for a long time or is now rare, (ii) a negative history of animal bites or exposure to rabies, and (iii) atypical clinical presentation of the disease. Clinical symptomatology of rabies is complex and commonly confuses physicians. Furthermore, failure in diagnosing imported cases in more developed countries is most likely related to the lack of medical familiarity with even the typical clinical features of the disease.

## Author Summary

Rabies is readily diagnosed when it presents in the classic furious form. The paralytic and atypical forms can pose significant problems in diagnosis, particularly when found in rabies-free countries in travelers who acquired the disease abroad. We systematically reviewed the existing literature and collected 60 cases of rabies in travelers and expatriates from non-endemic countries and in migrants from endemic countries during their first migration trip or following subsequent trip to their origin country. We observed that the disease may have an atypical presentation and/or long incubation time resulting in delayed or missed diagnosis with adverse public health consequences including multiple transmissions of rabies via transplanted solid organs from a single infected donor whose diagnosis of rabies was retrospectively assessed or need for risk assessment in a large number of patient contacts. Rabies should be suspected, even when a history of animal bites is missing, in patients with encephalitis or paralysis who originate or return from rabies-enzootic countries, notably in male adult patients, in migrants visiting countries of origin, and in the context of travel to India or the Philippines. The analysis of three serially collected saliva samples and one skin biopsy taken from the nape of the neck offers the highest level of sensitivity when using appropriate molecular techniques for viral RNA detection.

## Introduction

Rabies is readily diagnosed when it presents in the classic furious form. The paralytic and atypical forms can pose significant problems in diagnosis, particularly when found in rabies-free countries in travelers who acquired the disease abroad. The discussion of geographical and post-exposure prophylaxis issues of travel-associated human rabies has focused on preventive measures including pre-travel vaccination but has been limited to certain regions [1], [2]. An analysis of the compiled cases of rabies in travelers from a clinician's perspective is therefore absent. Since reports concerning these cases have been published, new cases have been documented and published. In this work, we present an updated analysis of travel-associated cases of human rabies with the objective of identifying potential risk factors and describing the procedures of clinicians with the aim of highlighting potential problems in the diagnosis and management of patients.

## Materials and Methods

To retrieve information on human rabies cases in travelers, we first conducted a literature search using the PubMed (MEDLINE) and Scopus databases (http://www.ncbi.nlm.nih.gov/pubmed; www.scopus.com/scopus/home.url), from 1980 to December 2012, cross-referencing the following terms: “rabies”, “imported” and “travel”. Relevant systematic and narrative reviews were also utilized to obtain useful background information. The reference lists of the systematic reviews and other identified papers were scanned for potentially relevant primary studies that could be considered for inclusion in the review. Additional searches were conducted using the ProMED-mail (http://www.promedmail.org/), Google (http://www.google.fr/), and Yahoo (http://fr.yahoo.com/) general search engines. Miscellaneous articles from Rabies Bulletin Europe were systematically scanned (www.scopus.com/scopus/home.url).

The inclusion criteria were all available publications written in European languages on human rabies cases in individuals who crossed a national border between the times of infection and diagnosis. Reports with insufficient clinical description were only included in the epidemiological analyses.

## Results

### Epidemiology

**Figure 1:**
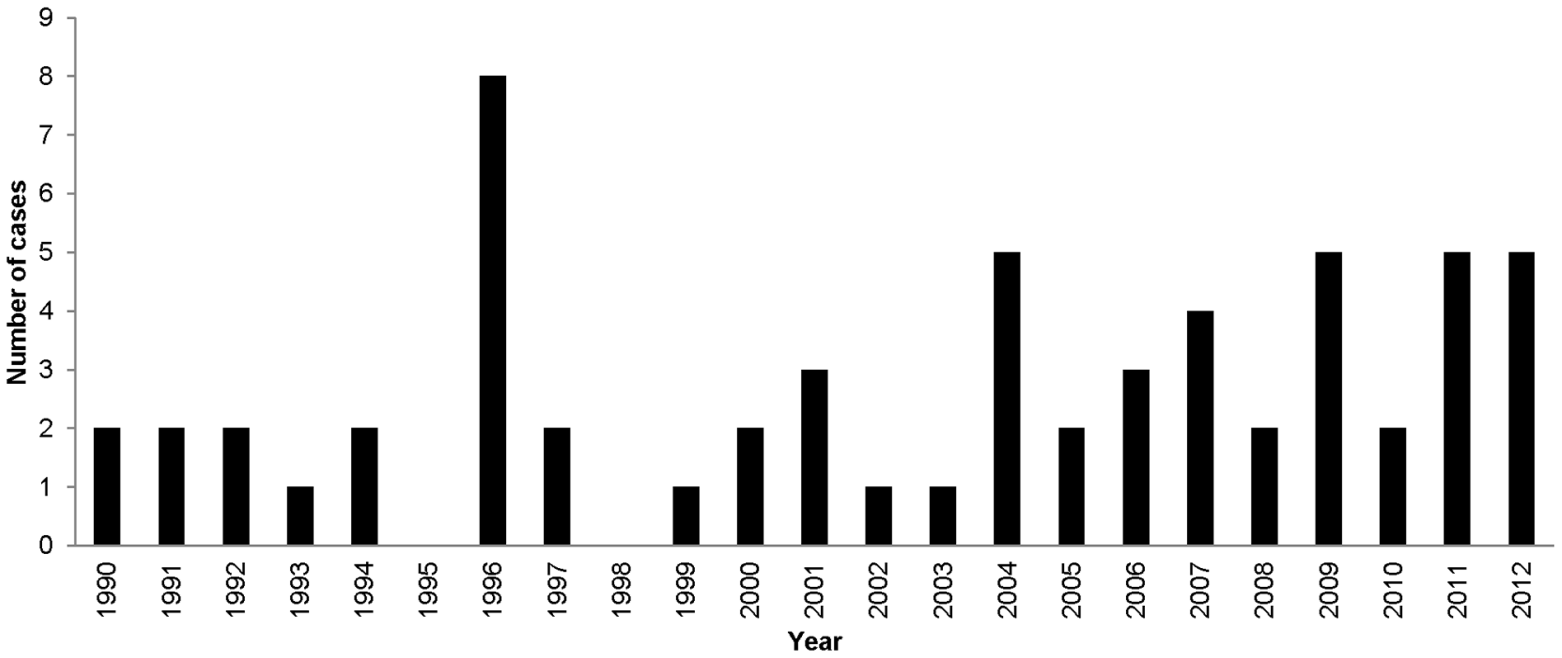
Number of human rabies cases in travelers per year (60 cases, 1990–2012).

**Figure 2:**
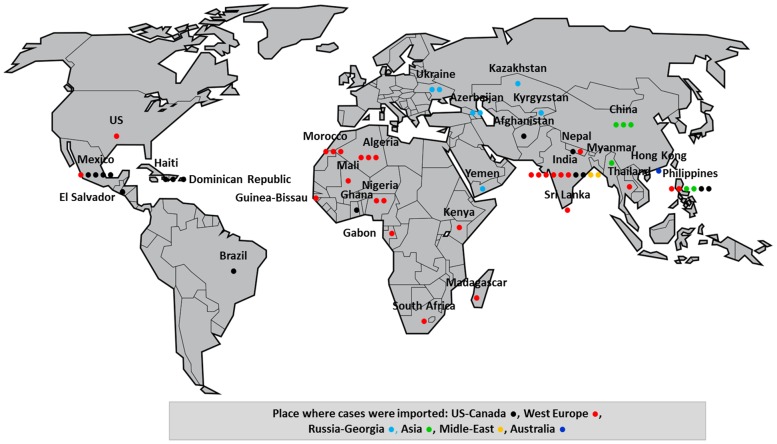
Country of exposure and place where the case was imported for 60 human rabies cases in travelers (1990–2012). Country of exposure: circle placed in, place where the cases was imported: according to color of circle.

**Figure 3:**
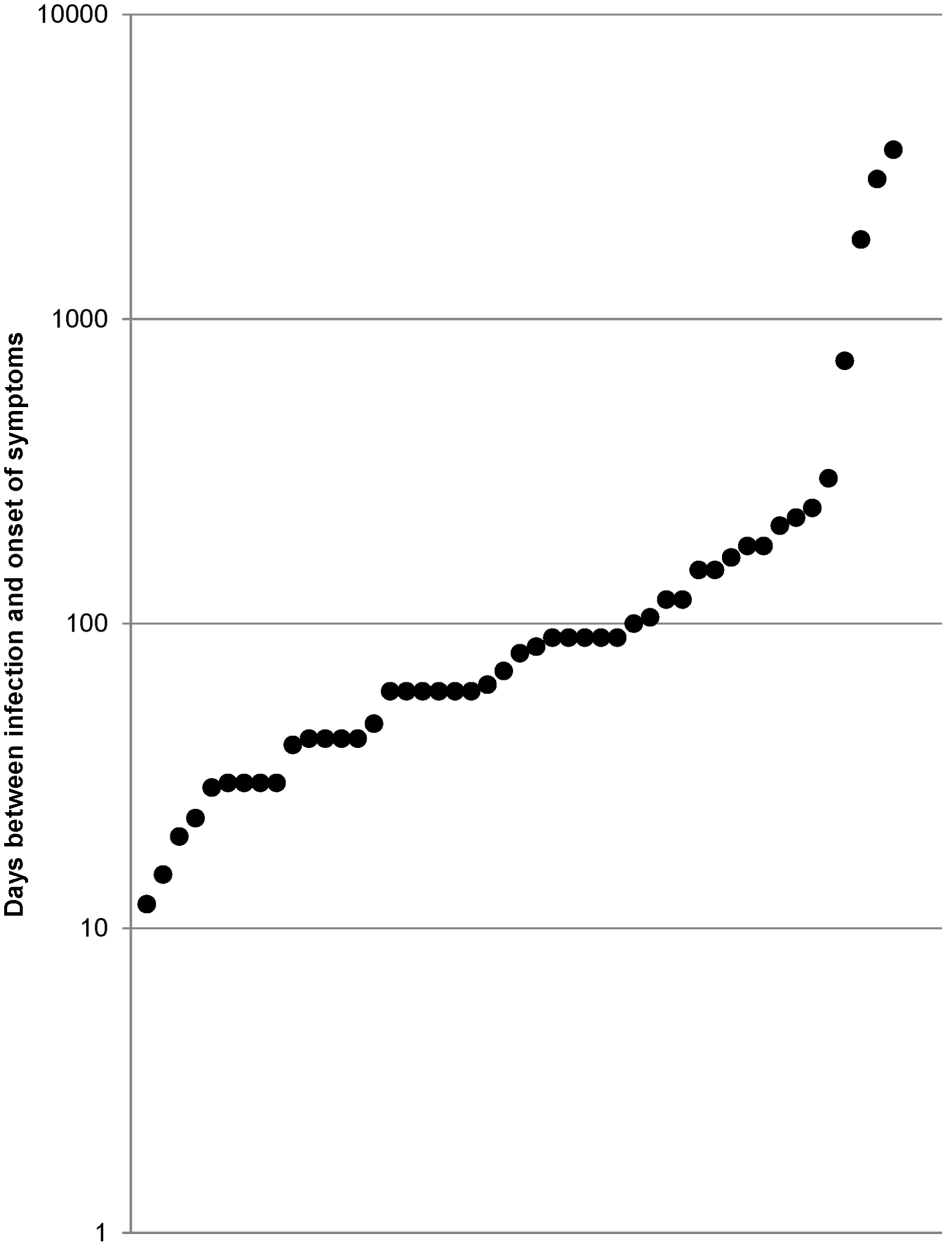
Incubation time (47 human rabies cases in travelers (1990–2012). Logarithmic scale. Each circle represents one patient.

Sixty cases met the inclusion criteria (see Supporting Text S1). The epidemiological data are summarized in figures 1 and 2. The description of travelers, data on clinical findings, laboratory results, diagnosis methods and treatments are shown in tables 1 to 3 and figure 3. An average of 2.6 cases were documented per year over the 23 years of the study with a slight increase from 1990–2003 to 2004–2012 (1.9 to 3.7 cases per year). The mean age of the patients was 37.7 years (range, 3–73 years) and the ratio of males to females was 3.5. Most cases were diagnosed in Europe (56.7%), notably in France (eight cases) and the United Kingdom and Ireland (six cases), and in the US (26.7%, n = 16). High income countries accounted for 56.7% of the cases in individuals travelling for tourism, business or expatriation. Migrants originating from low income countries and their descendants accounted for 43.3% of cases when taking their first trip abroad, visiting friends and relatives in their country of origin, or traveling to seek care, for business or for other undocumented reasons. Most exposures occurred in Asia (40.0%), notably in India (10 cases) and the Philippines (six cases); in Central America and the Caribbean (13.3%), notably in Mexico (five cases); and in North Africa (10.0%), notably in Morocco and Algeria (six cases). Travel duration was not documented in the majority of the reports. Two cases were recorded in tourists taking two-week trips to India and Kenya. The vast majority (85.0%) of cases resulted from exposure to dogs. Three cases resulted from bat-related injuries, including one case of a Dutch tourist returning from Kenya [3]–[5], one case of a US citizen injured in the US who developed rabies symptoms while expatriated in Iraq and was subsequently evacuated to Switzerland [6], and one case of a Mexican citizen who developed rabies in the US where he was involved in seasonal work [7], [8]. One case was observed in Russia following a fox bite that occurred in Ukraine [2].

**Table 1:** Demographics, travel characteristics and source of exposure for 60 travel-associated human rabies cases (1990–2012).

Category	Subcategory	N (%)
Age	≤5 years	3 (5.0)
	5–15 years	4 (6.7)
	16–59 years	42 (70.0)
	≥60 years	8 (13.3)
	Not documented	3 (5.0)
Gender	Male	45 (75.0)
	Female	13 (21.7)
	Not documented	2 (3.3)
Place of residence	Europe^1^	30 (50.0)
	US-Canada^2^	13 (21.7)
	Asia^3^	7 (11.7)
	Latin America^4^	4 (6.7)
	Middle East^5^	3 (5.0)
	Africa^6^	2 (3.3)
	Australia	1 (1.7)
Reason for travel	Visiting friends and relatives^7^	14 (23.3)
	Tourism	8 (13.3)
	Migration (from low to high income country)	7 (11.7)
	Expatriation (from high to low income country)	5 (8.3)
	Business	5 (8.3)
	Medical evacuation	2 (3.3)
	Military	1 (1.7)
	Volunteer work	1 (1.7)
	Not documented	17 (28.3)
Source of infection	Dog	51 (85.0%)
	Bat	3 (5.0%)
	Fox	1 (1.7%)
	Unknown animal or not documented	5 (8.3%)

1 France: 7, Russia and other former USSR countries: 7, United Kingdom and Ireland: 5, Germany: 4, The Netherlands: 2, Italy; 2, Austria: 1, Portugal: 1, Sweden: 1.

2 US: 12, Canada: 1.

3 China: 3, Japan: 2, Philippines: 1, Myanmar: 1.

4 Mexico: 3, Haiti: 1.

5 Israel: 1, United Arabian Emirates: 1, Iraq: 1.

6 Algeria: 1, Nigeria: 1.

7 non-recent migrants or their descendants visiting friends and relatives in their origin countries.

**Table 2:** Clinical and microbiological features of 60 travel-associated human rabies cases (1990–2012).

Category	Subcategory	N (%)
History of animal bite at presentation	Yes	21 (35.0)
	No	25 (41.7)
	Not documented	14 (23.3)
Number of health care providers consulted before diagnosis of rabies was made	1	13 (21.7)
	2	13 (21.7)
	3	8 (13.3)
	4	8 (13.3)
	≥5	2 (3.3)
	Not documented	16 (26.7)
Clinical form^1^	Furious	45 (75.0)
	Paralytic	6 (15.0)
	Not documented	9 (10.0)
Biological confirmation of rabies	Ante-mortem	28 (46.7)
	Post-mortem	20 (33.3)
	Not documented	12 (20.0)
Methods allowing biological confirmation of rabies^2^	RTPCR, salivary gland or saliva	16 (26.7)
	RTPCR, skin	10 (16.7)
	RTPCR, brain	
	RTPCR, cerebrospinal fluid	2 (3.3)
	RTPCR, conjunctival swab	1 (1.7)
	RTPCR, throat swab	1 (1.7)
	FAT, brain	13 (21.7)
	FAT, skin	9 (15.0)
	Serology, serum and/or cerebrospinal fluid	7 (11.7)
	Virus isolation in mouse brain cells, saliva	3 (5.0)
	Not documented	14 (23.3)

1 Furious form : classic febrile encephalitic form where signs of irritation of the central nervous system predominate, including agitation, confusion, hydrophobia and aerophobia; paralytic form : paralysis involving the peripheral nerves, usually accompanied by fever.

2 The sum of percentages is over 100% because rabies was confirmed by more than one method in a number of patients.; RTPCR = reverse transcriptase polymerase chain reaction; FAT = fluorescent antibody test.

**Table 3:** Treatment in 60 travel-associated human rabies cases (1990–2012).

Category	Subcategory	N (%)
Rabies post-exposure prophylaxis in country of exposure	None	43 (71.7)
	Rabies vaccine^1^	5 (8.3%)
	Not documented	12 (20.0)
Rabies treatment attempt in country of diagnosis^2^	None	29 (48.3)
	Rabies vaccine only	2 (3.3)
	Rabies vaccine+rabies immune globulin	5 (8.3)
	Rabies immune globulin only	1 (1.7)
	Rabies immune globulin+Milwaukee protocol	1 (1.7)
	Milwaukee protocol^3^	6 (10.0)
	Not documented	16 (26.7)

1 Incomplete course in 2 cases.

2 Treatment was initiated after the onset of symptoms in all cases.

3 induction of coma with pentobarbital, midazolam and ketamine and use of antivirals amantadine and ribavirin.

### Incubation time

The incubation time was documented in 47/60 records (Figure 3) with a mean incubation time of 273.6 days (median time: 80 days, range, 12–3600 days), including nine cases with an incubation time of 30 days or less. Very short incubation times were observed in two cases. A 50-year-old French female tourist sustained multiple deep dog bites on the legs during a trip in India and developed rabies 12 days later while returning to France [9], [10]. A 19-year-old male Mexican seasonal worker was bitten by a bat on his leg and developed rabies due to a variant virus of vampire bat rabies 15 days later in the US [7], [8]. In three cases, very long incubation times were recorded. A 10-year-old female migrant from Vietnam who stayed 1.5 years in Hong Kong prior to immigrating to Australia developed rabies more than five years after she had lived continuously in Australia. The sequence of the rabies virus isolated post-mortem was closely related to a subgroup of viruses found in China. No history of animal bites was documented [11]–[13]. A 40-year-old man developed rabies in the US due to a canine rabies virus variant associated with dogs in Brazil, which was identified by sequence analysis of viral amplicons. After the diagnosis was established, interviews with family members indicated a history of contact with a “rabid-acting” dog while living in Brazil, approximately 8 years prior to becoming ill. An investigation of the patient's travel history did not identify any intermittent travel to Brazil since that time [14]. An 18-year-old male recent migrant, originating from Myanmar, developed rabies in Thailand. He gave a history of dog bites 10 years before, and he denied any recent animal bites or contact with bats [15].

### Clinical features and biological confirmation of rabies

In 46 records, information about whether or not a history of animal bite was investigated at initial presentation was available. Only 26/46 (56.2%) patients reported a history of animal bite at first medical encounter. Number of health care provider consulted before a diagnosis of rabies was made was available in 44 records. 31/44 (70.5%) patients consulted several health care providers before a diagnosis of rabies was obtained. In these patients, an incorrect primary diagnosis was given including acute psychiatric illness, anxiety, depression, influenza like illness, meningitis, cervical radiculopathy, Guillain-Barré syndrome, Bickerstaff's encephalitis, angina pectoris, pharyngitis, lumbago, and constipation. In 45/60 (75.0%) patients the acute neurological signs were furious while they were paralytic in 6/60 (15.0%); the clinical features were not documented in nine records. Among patients with furious form of rabies, 11/45 (24.4%) received a diagnosis of rabies at first medical consultation of which 9/11 (81.8%) reported a history of animal bite, 27/45 (60.0%) consulted several health care providers before a diagnosis of rabies was made, of which 8/27 (29.6%) reported a history of animal bite at first medical encounter. The number of health care providers consulted was not documented in 7 records. Most cases resulted from bites inflicted by dogs, and in 26 patients whose information was available, wounds were located on the upper limbs in most cases. A confirmed diagnosis of rabies was obtained post-mortem in one-third of the cases. In 4 instances, a diagnosis of rabies was only considered after death, including one case in an organ donor whose death was not considered to be related to rabies at the time of death. Overall, the mean time between hospitalization and suspected diagnosis of rabies based on clinical features was 3.9 days (median time: one day, range, 1–30). The time between hospitalization and a confirmed diagnosis of rabies was documented in 27 out of 28 patients whose diagnosis was confirmed ante-mortem. The mean time was 7.7 days (median time: 6.0 days, range 2–30), including four cases with diagnoses delayed by 15, 16, 18 and 30 days [7], [8], [14], [16]–[18]. Reasons for delayed diagnosis were an absence of a history of animal bites at presentation in three cases and a paralytic form mimicking Guillain-Barré syndrome in three cases. In patients whose rabies diagnosis was confirmed post-mortem, a suspected diagnosis of rabies was delayed by 22 days because of the absence of a history of animal bites at presentation [6]. Hydrophobia and aerophobia were present in 60.0% and hyper-salivation was present in 78.6% of the patients whose clinical records were available. Several atypical cases were described, including a UK tourist with a history of dog bites in India and an initial presentation of lower back pain radiating in the leg. The patient was first hospitalized in orthopedics, and then referred to a medical ward where a provisional diagnosis of Guillain-Barré syndrome was made because of the appearance of flaccid weakness in both legs and arms. A few days later, due to absent oculocephalic reflexes and unreactive pupils, a diagnosis of Bickerstaff's encephalitis was considered. Subsequently, the infectious diseases unit and specialist neurology center were contacted for advice, and rabies was suspected [16], [17]. A Nigerian visitor to the UK who developed fever, exhibited altered behavior, and manifested malarial parasites in blood films was diagnosed with cerebral malaria before rabies was diagnosed post-mortem [19]–[22]. A migrant from Myanmar presented in Bangkok, Thailand with fever and dysphagia. There was a history of fluctuating consciousness and aerophobia, but they were absent or could not be demonstrated at the time of admission. He exhibited subcutaneous chest wall emphysema and was found to have pneumomediastinum, which resulted in surgical intervention. He developed paralysis followed by seizures during the postoperative period. Diagnosis was confirmed during the preterminal phase [15].

Among the 60 cases presented in this review, confirmation of rabies was assessed by reverse transcriptase polymerase chain reaction (RTPCR) in most cases, primarily from salivary gland biopsy or saliva and skin biopsy. Computed tomography scans of the brain were reported in 16 cases, none of which contributed to the diagnoses. Cerebral magnetic resonance imaging (MRI) was reported in 14 cases of which three showed typical alterations: high signal intensity on T2-weighted images bilaterally in the hippocampal gyri and the head of the caudate nucleus in one case [16], [17]; hyperintense signal changes bilaterally in the caudate nucleus, thalamus, mesencephalon, pons and medulla oblongata in the second case [22]–[25] and T2-weighted images in the posterior part of the medulla oblongata and pons in the third case [3]–[5]. In these three cases, the diagnosis of rabies was assessed before the MRIs were obtained.

### Treatment and outcome

Rabies post-exposure prophylaxis was provided in the country of infection in less than 8% of cases. Interestingly, two cases were recorded in medical doctors who did not receive post-exposure prophylaxis following animal-related injuries [3]–[5], [26]. All therapeutic attempts that were conducted after the onset of disease were unsuccessful, including seven patients who underwent the experimental “Milwaukee” protocol including induction of coma with pentobarbital, midazolam and ketamine and the use of the antivirals amantadine and ribavirin [3]–[5], [26]–[33]. The mean time between hospitalization and death was 14.4 days (median time: 12.0 days, range, 1–40) with no significant differences between those who underwent the “Milwaukee” protocol and those who did not.

## Discussion

It is very likely that a number of confirmed travel-associated rabies cases were unpublished or published in journals not indexed in PubMed and Scopus. Moreover, rabies may be misdiagnosed, notably when death occurs abroad. The figure of two-four cases per year of travel-associated cases of rabies is most an underestimate of true incidence. Risk factors cannot be extrapolated from our results because we lack denominators. However, several points need to be stressed: (i) a significant proportion of cases were observed in migrants or their descendants when emigrating from their country of origin or following a trip with the purpose of visiting friends and relatives (43.3%), (ii) cases were not necessarily associated with long-term travel or expatriation to endemic countries; rather, cases were observed in travelers undergoing short trips of 2 weeks or less, (iii) a predominance of male patients was observed (75.0%), (iv) children, although typically accounting for a large proportion of cases in people living in rabies-endemic areas were rare among travelers (11.7%), (v) cases from India and the Philippines were frequent (16 cases/60), and (vi) 85% of cases had dog as a source of infection.

An important finding of this study concerning rabies in travelers is that in a significant proportion of cases, diagnosis was challenging with multiple missed diagnoses and transfers from ward to ward before a diagnosis of rabies was finally assessed or clinical diagnosis was delayed (more than seven days post-hospitalization) with post-mortem biological confirmation and/or delayed ante-mortem biological diagnosis (more than seven days post-hospitalization). In five cases, patients traveled through one or more countries before ultimately being hospitalized. The diagnosis was challenging in 24 out of 47 cases (51.1%) in which such information was available. Three factors played a role in delaying the diagnosis of rabies in a number of cases: (i) a low index of suspicion for rabies in countries where the disease has been eradicated for a long time or is now rare, (ii) a negative history of animal bites or exposure to rabies, and (iii) atypical clinical presentation of the disease including paralytic form, and furious form initially mimicking sore-throat infection, orthopedic or acute psychiatric disorder. A delayed diagnosis of rabies can have adverse public health consequences including multiple transmissions of rabies via transplanted solid organs from a single infected donor whose diagnosis of rabies was retrospectively assessed [22], [34]–[36]. It may also lead to the need for risk assessment in a large number of patient contacts, as recently exemplified in Louisiana where 204 individuals were investigated by public health officials and hospital infection control staff, resulting in 95 requiring post-exposure prophylaxis, including 68 healthcare workers [7], [8]. In the present work, we observed a large heterogeneity in the use of laboratory techniques, which was due to the length of the study period and differences between countries. The clinical symptomatology of rabies is complex and commonly causes confusion to physicians. Furthermore, failure to diagnose imported cases in more developed countries is likely to be related to the lack of medical familiarity with even the typical clinical features of the disease.

Rabies should be suspected, even when a history of animal bites is missing, in patients with encephalitis or paralysis who originate or return from rabies-enzootic countries, notably in male adult patients, in migrants visiting countries of origin and in the context of a travel to India or the Philippines. The analysis of three serially collected saliva samples and one skin biopsy taken from the nape of the neck offers the highest level of sensitivity when using appropriate molecular techniques for viral RNA detection [37].

Rabies can be efficiently averted by preexposure immunization, avoidance of contact with animals, and postexposure prophylaxis (PEP). Preventive vaccination against rabies has to be considered for travelers to rabies endemic areas because it simplifies PEP from five vaccine doses over 28 days to two vaccine doses over three days, and it eliminates the need for immunoglobulin administration. In the present work, many cases were associated with short duration travel which challenges the common view that preventive vaccination against rabies should be preferentially given to long-term travelers to high risk areas. Many of the rabies cases were in migrants traveling to their origin country who may lack the budget for pre-travel vaccination. The intradermal vaccination route has been proven economical, safe, and immunogenic in the population of rabies-endemic areas, and this route of administration has been recently used in travelers from developed countries [1], [38]. Alternatively, initial short schedules induce rapid and sufficient antibody responses provided that a booster vaccination is provided [39]. The immunity provided by the gold-standard three-dose series is long-lasting and can be maintained over at least a decade, thus preventive vaccination against rabies should be considered an investment for future travel [38].

## Supporting Information

Text S1 60 travel-associated human rabies cases-references.
(DOCX)
