# Supplementary material for: Imported Human Rabies Cases Worldwide, 1990–2012
Source: PLoS Negl Trop Dis. 2013 May 2;7(5):e2209. doi: 10.1371/journal.pntd.0002209 (PMC3642086; doi:10.1371/journal.pntd.0002209)
Supplement: Text S1 — 60 travel-associated human rabies cases-references. (DOCX) [file pntd.0002209.s001.docx]

**Supporting Text S1 - 60 travel-associated human rabies cases-references.**

| **Year and country where the case was imported^1^** | **Country of exposure** | **References** |
| --- | --- | --- |
| ***Europe***  **Austria** (illness onset in Ceuta, Spain)  2004 | Morocco | 1 |
| **Finland**  2007 | Philippines | 2 |
| **France**  1990  1992  1994  1996 (Reunion Island)  1996  1996  1997  2003 | Mexico  Algeria  Mali  Madagascar  Algeria  Algeria  India  Gabon | 3-7  4,6-9  7,10  7,10  7,10  7,10-12  7,10  7,13,14 |
| **Georgia**  2010 | Azerbaijan | 15 |
| **Germany**  1996  2004  2004  2007 | Sri Lanka  India  India  Morocco | 16,17  18-21  21-24  25,26 |
| **Italy**  1996  2011 (illness onset in Germany) | Nepal  India | 27  28 |
| **Portugal**  2011 | Guinea Bissau | 29 |
| **Russia**  1991  1991  2007  2009  2009  2009 | Ukraine  Yemen  Ukraine  Azerbaijan  Kazakhstan  Azerbaijan | 30  30  31  31  31  31 |
| **Sweden**  2000 | Thailand | 32 |
| **Switzerland (**illness onset in Thailand)^1^  2012 | US | 33 |
| **The Netherlands**  1997  2007 | Morocco  Kenya | 34,35  36-38 |
| **United Kingdom and Ireland**  1996 (England)  2001 (England)  2001 (England)  2005 (England)  2008 (Ireland)  2012 (England) | Nigeria  Nigeria  Philippines  India  South Africa  India | 21,39-41  21,42,43  21,43,44  21,45,46  47,48  49-51 |
| ***US-Canada***  1992 (California)  1993 (California)  1994 (Miami)  1996 (Florida)  1996 (New Hampshire, illness onset in Thailand)^2^  2000 (New York)  2001 (California)  2004 (California)  2004 (Florida)  2006 (California)  2008 (California)  2009 (Virginia)  2010 (Louisiana)  2011 (New Jersey)  2011 (New York, illness onset during travel from Germany to the US)  2011 (Massachusetts)  2012 (Canada) | India  Mexico  Haiti  Mexico  Nepal  Ghana  Philippines  Salvador  Haiti  Philippines  Mexico  India  Mexico  Haiti  Afghanistan  Brazil  Dominican Republic | 52  53  54  55  56  57  58  59  60  61  62  63  64,65  66  67  68  69 |
| ***Asia***  **Japan**  2006  2006  **Singapore**  1999  **Taiwan**  2002  2012  **Thailand**  2005 | Philippines  Philippines  China  China  China  Myanmar | 70,71  70,72  73  74,7475  76  77 |
| ***Middle East***  **UAE**  2009  **Israel**  2012 (onset of symptoms in China) | India  India | 78  79 |
| **Australia**  **1990** | Hong Kong | 80-82 |

^1^California resident, expatriate in Iraq, experienced first symptoms in Thailand during leisure trip, returned to Iraq and was then hospitalized in Dubai, United Arabian Emirates and subsequently transferred to Zurich, Switzerland at his family’s request.

^2^ traveled to Thailand and then to Australia to seek post-exposure prophylaxis unsuccessfully, before returning to US.

**References**

1. Strauss R, Granz A, Wassermann-Neuhold M, Krause R, Bagó Z, Revilla-Fernández S, Simón-Soria FS, Echevarría JE, Popow-Kraupp T, Allerberger F, Schönbauer M, Hrabcik H. A human case of travel-related rabies in Austria, September 2004. Euro Surveill 2005; 10:225–226.

2. Rimhanen-Finne R, Järvinen A, Kuusi M, Quiambao BP, Malbas FF Jr, Huovilainen A, Kallio-Kokko H, Vapalahti O, Ruutu P. Imported human rabies, the Philippines and Finland, 2007. Emerg Infect Dis 2010; 16:1318-9.

3. Rollin P. Imported human rabies – France. Rabies Bulletin Europe 1991; 15/(1): 11.

4. Rollin P, Sureau P. Le point sur la prophylaxie de la rage humaine en France en 1991. Bull Epid Hebdo. 1992; 30:141-2.

5. Hofman P, Bourhy H, Michiels JF, Dellamonica P, Sureau P, Boissy C, Loubière R. Rabies encephalomyelitis with myocarditis and pancreatitis. Report on a case recently imported into France. Ann Pathol. 1992; 12:339-46.

6. World Health Organization (WHO). Rabies. Two cases of imported rabies in France. Wkly Epidemiol Rec. 1992; 67:361-2.

7. Peigue-Lafeuille H, Bourhy H, Abiteboul D, Astoul J, Cliquet F, Goudal M, Lerasle S, Mailles A, Montagne MC, Morer I, Rotivel Y, Floret D. Human rabies in France in 2004: update and management. Med Mal Infect. 2004; 34:551-60.

8. Centers for Disease Control (CDC). Imported human rabies--France, 1992. MMWR Morb Mortal Wkly Rep. 1992; 41:953-5.

9. Sureau P, Herzog M, Bourhy H. A case of imported human rabies in France. Rabies Bulletin Europe 1992; 16(2): 9-10.

10. Rotivel Y, Bourhy H, Wirth S, Goudal M, Tsiang H. Imported human rabies cases in France. Rabies bulletin Europe. 1997; 21(4):14.

11. Fauquet S. La rage en France, à propos d’un cas marseillais. Thèse pour le doctorat en médecine, Marseille, 1996.

12. Gautret P, Adehossi E, Soula G, Soavi MJ, Delmont J, Rotivel Y, Brouqui P, Parola P. Rabies exposure in international travelers: do we miss the target? Int J Infect Dis. 2010; 14: e243–e246.

13. Editorial team. Human case of rabies in a child in France who had visited Gabon. Euro Surveill. 2003; 7(46): pii=2327.

14. Groupe de travail du Conseil supérieur d'hygiène publique de France. Recommandations relatives à la conduite à tenir dans l'entourage d'un cas de rage humaine. 18 juin 2004.

15. ProMED-mail. Published Date: 2010-12-22 18:00:05. Rabies, human - Georgia, and ex Azerbaijan. Archive Number: 20101222.4507.

16. Müller WW. Imported human rabies case in Germany. Rabies Bulletin Europe 1996;

17. Roß RS, Kruppenbacher JP, Schiller WG, Marcus I, Kirsch WD, Wiese M, Adamczak M, Roggendorf M. Menschliche Tollwuterkrankungen in Deutschland. Deutsches Ärzteblatt 1997; 94, A34-A37.20(2): 9-10.

18. Robert Koch-Institut. Tollwut – ein Erkrankungsfall nach Indienaufenthalt. Robert Koch-Institut Epidemiologisches Bulletin 2004; 42:362-3.

19. Summer R, Ross S, Kiehl W. Imported case of rabies in Germany from India. Euro Surveill. 2004; 8(46):pii=2585.

20. Schankin CJ, Birnbaum T, Linn J, Brüning R, Kretzschmar HA, Straube A, Krebs B. A fatal encephalitis. Lancet. 2005; 365:358.

21. Johnson N, Brookes SM, Fooks AR, Ross RS. Review of human rabies cases in the UK and in Germany. Vet Rec. 2005; 157:715.

22. Robert Koch-Institut. Informationen zu den Tollwutübertragungen durch Spenderorgane. Robert Koch-Institut Epidemiologisches Bulletin 2005; 8:70.

23. Hellenbrand W, Meyer C, Rasch G, Steffens I, Ammon A. Cases of rabies in Germany following organ transplantation. Euro Surveill. 2005; 10(8):pii=2917.

24. Maier T, Schwarting A, Mauer D, Ross RS, Martens A, Kliem V, Wahl J, Panning M, Baumgarte S, Müller T, Pfefferle S, Ebel H, Schmidt J, Tenner-Racz K, Racz P, Schmid M, Strüber M, Wolters B, Gotthardt D, Bitz F, Frisch L, Pfeiffer N, Fickenscher H, Sauer P, Rupprecht CE, Roggendorf M, Haverich A, Galle P, Hoyer J, Drosten C. Management and outcomes after multiple corneal and solid organ transplantations from a donor infected with rabies virus. Clin Infect Dis. 2010; 50:1112-9.

25 Schmiedel S, Panning M, Lohse A, et al. Case report on fatal human rabies infection in Hamburg, Germany, March 2007. Euro Surveill 2007; 2007; 12:pii=3210.

26. Drosten C, Schmiedel S, Panning M, Burchard G, Gerloff C, Lohse A. Human Rabies in Europe in the 1st quarter 2007 – case studies. Germany. Rabies Bulletin Europe 2007; 31(1): 5-6.

27. Bechi M, Bernardi D, Scarpa M, Muttinelle F, Tollot M, Raise E. Rabbia umana di importazione, descrizione di un caso e note epidemiologiche. Giorn It Mal Inf 1996; 5:305-7.

28. De Benedictis P, Perboni G, Gentili C, Gaetti L, Zaffanella F, Mutinelli F, Capua I, Cattoli G. Fatal case of human rabies imported to Italy from India highlights the importance of adequate post-exposure prophylaxis, October 2011. Euro Surveill. 2012;17 :pii: 20168.

29. Santos A, Cale E, Dacheux L, Bourhy H, Gouveia J, Vasconcelos P. Fatal case of imported human rabies in Amadora, Portugal, August 2011. Euro Surveill. 2012;17:pii: 20130.

30. Cherkasskiy BL, Khairushev AE, Knop AG. Human rabies in the European part of Russia in 1991. Rabies Bulletin Europe 1992; 16(1):15.

31. Malerczyk C, Detora L, Gniel D. Imported human rabies cases in Europe, the United States, and Japan, 1990 to 2010. J Travel Med. 2011; 18:402-7.

32. Höjer J, Sjöblom E, Berglund O, Hammarin AL, Grandien M. The first case of rabies in Sweden in 26 years. Inform travelers abroad about risks and treatment following suspected infection. Lakartidningen. 2001; 98:1216-20.

33. Centers for Disease Control and Prevention (CDC). U.S-acquired human rabies with symptom onset and diagnosis abroad, 2012. MMWR Morb Mortal Wkly Rep. 2012; 61:777-81.

34. Schrijver HM, Veering MM, Vis MM. A patient with rabies in The Netherlands. Ned Tijdschr Geneeskd. 1997; 141:437-9.

35. Groen J, Veering MM, Leentvaar-Kuipers A, OsterhausAD. A case of human rabies in the Netherlands. Infection1998; 26:196.

36. van Thiel PP, van den Hoek JAR, Eftimov F, Tepaske R, Zaaijer HJ, Spanjaard L, de Boer HEL 7, van Doornum GJJ, Schutten M, Osterhaus ADME, Kager PA. Fatal case of human rabies (Duvenhage virus) from a bat in Kenya: the Netherlands, December 2007. Rabies Bulletin Europe 2007; 31(4): 5-6.

37. van Thiel PP, van den Hoek JA, Eftimov F, Tepaske R, Zaaijer HJ, Spanjaard L, de Boer HE, van Doornum GJ, Schutten M, Osterhaus A, Kager PA. Fatal case of human rabies (Duvenhage virus) from a bat in Kenya: the Netherlands, December 2007. Euro Surveill. 2008; 13:e428.

38. van Thiel PP, de Bie RM, Eftimov F, Tepaske R, Zaaijer HL, van Doornum GJ, Schutten M, Osterhaus AD, Majoie CB, Aronica E, Fehlner-Gardiner C, Wandeler AI, Kager PA. Fatal human rabies due to Duvenhage virus from a bat in Kenya: failure of treatment with coma-induction, ketamine, and antiviral drugs. PLoS Negl Trop Dis. 2009; 3:e428.

39. Public Health Laboratory Service (PHLS). A case of human rabies acquired in Nigeria. Comm Dis Rep Wkly. 1996; 6 (42):1.

40. World Health Organization (WHO). A case of human rabies contracted in Nigeria. Wkly Epidemiol Rec. 1997; 22: 163-164.

41. Anonymous, A case of human rabies contracted in Nigeria. Can Commun Dis Rep. 1997; 23:151.

42. Johnson N, Lipscomb DW, Stott R, Gopal Rao G, Mansfield K, Smith J, McElhinney L, Fooks AR. Investigation of a human case of rabies in the United Kingdom. J Clin Virol. 2002; 25:351-6.

43. Fooks AR, Johnson N, Brookes SM, Parsons G, McElhinney LM. Risk factors associated with travel to rabies endemic countries. J Appl Microbiol. 2003; 94 Suppl:31S-36S.

44. Smith J, McElhinney LM, Parsons G, et al. Case report: rapid antemortem diagnosis of a human case of rabies imported into the UK from the Philippines. J Med Virol 2003; 69:150-5.

45. Solomon T, Marston D, Mallewa M, Felton T, Shaw S, McElhinney LM, Das K, Mansfield K, Wainwright J, Kwong GN, Fooks AR. Paralytic rabies after a two week holiday in India. BMJ. 2005 Sep 3;331(7515):501-3.

46. Smith A, Petrovic M, Solomon T, Fooks A. Death from rabies in a UK traveller returning from India. Euro Surveill. 2005; 10(7):E050728.5.

47. Harkess G, Johnson N, Marston D, Goddard T, Goharriz H, Voller K, Solomon, T, Hunter M, Hedderwick S, McCaughey C, Willoughby R, Fooks, AR. Tracking the Infection in Real Time in a Fatal Case of Human Rabies. Rabies Bulletin Europe 2010; 34(1): 7-8.

48. Hunter M, Johnson N, Hedderwick S, McCaughey C, Lowry K, McConville J, Herron B, McQuaid S, Marston D, Goddard T, Harkess G, Goharriz H, Voller K, Solomon T, Willoughby RE, Fooks AR. Immunovirological correlates in human rabies treated with therapeutic coma. J Med Virol. 2010; 82:1255-65.

49. ProMED-mail. Published Date: 2012-05-24. Rabies - UK (02): (London, England) ex India, human, canine. Archive Number: 20120524.1143694.

50. ProMED-mail. Published Date: 2012-05-23. Rabies - UK: (England) ex India, human, canine. Archive Number: 20120523.1142429

51. ProMED-mail. Published Date: 2012-05-29 09:36:49. Rabies - UK (03): (London) ex India, fatal outcome. Archive Number: 20120529.1148757

52. Centers for Disease Control (CDC). Human rabies--California, 1992. MMWR Morb Mortal Wkly Rep. 1992; 41:461-3.

53. Centers for Disease Control and Prevention (CDC). Human rabies--Texas and California, 1993. MMWR Morb Mortal Wkly Rep. 1994; 43:93-6.

54. Centers for Disease Control and Prevention (CDC). Human rabies--Miami, 1994. MMWR Morb Mortal Wkly Rep. 1994; 43(42):773-5.

55. Centers for Disease Control and Prevention (CDC).Human Rabies—Florida, 1996. MMWR Morb Mortal Wkly Rep 1996; 45:719-20.

56. Centers for Disease Control and Prevention (CDC). Human rabies—New Hampshire, 1996. MMWR Morb Mortal Wkly Rep 1997; 46:267-70.

57. Centers for Disease Control and Prevention (CDC). Human rabies—California, Georgia, Minnesota, New York, and Wisconsin, 2000. MMWR Morb Mortal Wkly Rep 2000; 49:1111-15.

58. Krebs JW, Noll HR, Rupprecht CE, Childs JE. Rabies surveillance in the United States during 2001. J Am Vet Med Assoc. 2002; 221:1690-701.

59. Acute Communicable Disease Control (ACDC). Human rabies death in Los Angeles County: first human case in 30 years. Acute Communicable Disease Control. 2005 Special Studies Report. Pp 39-40.

60. Centers for Disease Control and Prevention (CDC). Human rabies—Florida, 2004. MMWR Morb Mortal Wkly Rep 2005; 54:767-8.

61. Centers for Disease Control and Prevention (CDC). Human rabies—Indiana and California, 2006. MMWR Morb Mortal Wkly Rep 2007; 56:361-5.

62. Centers for Disease Control and Prevention (CDC). Imported human rabies--California, 2008. MMWR Morb Mortal Wkly Rep. 2009; 58:713-6.

63. Centers for Disease Control and Prevention (CDC). Human rabies---Virginia, 2009. MMWR Morb Mortal Wkly Rep. 2010; 59:1236-8.

64. Centers for Disease Control and Prevention (CDC). Human rabies from exposure to a vampire bat in Mexico --- Louisiana, 2010. MMWR Morb Mortal Wkly Rep. 2011;60:1050-2.

65. Mader EC Jr, Maury JS, Santana-Gould L, Craver RD, El-Abassi R, Segura-Palacios E, Sumner AJ. Human rabies with initial manifestations that mimic acute brachial neuritis and Guillain-Barré syndrome. Clin Med Insights Case Rep. 2012; 5:49-55.

66. Centers for Disease Control and Prevention (CDC). Imported human rabies –New Jersey, 2011. MMWR Morb Mortal Wkly Rep 2012; 60:1734-36

67. Centers for Disease Control and Prevention (CDC). Imported human rabies in a U.S. Army soldier – New York, 2011. MMWR Morb MortalWkly Rep 2012; 61:302-5.

68. Blanton JD, Dyer J, McBrayer J, Rupprecht CE. Rabies surveillance in the United States during 2011. J Am Vet Med Assoc. 2012; 24:712-22.

69. ProMED-mail. Published Date: 2012-04-17 22:57:49. Rabies - Canada (ON): human, ex Dominican Republic. Archive Number: 20120417.1104531.

70. Tamashiro H, Matibag GC, Ditangco RA, Kanda K, Ohbayashi Y. Revisiting rabies in Japan: is there cause for alarm? Travel Med Infect Dis. 2007; 5:263-75.

71. Yamamoto S, Iwasaki C, Oono H, Ninomiya K, Matsumura T. The first imported case of rabies into Japan in 36 years: a forgotten lifethreatening disease. J Travel Med. 2008; 15:372-4.

72. Tobiume M, Sato Y, Katano H, Nakajima N, Tanaka K, Noguchi A, Inoue S, Hasegawa H, Iwasa Y, Tanaka J, Hayashi H, Yoshida S, Kurane I, Sata T. Rabies virus dissemination in neural tissues of autopsy cases due to rabies imported into Japan from the Philippines: immunohistochemistry. Pathol Int. 2009; 59:555-6.

73. Oon CT. Boerhaave's syndrome (ruptured oesophagus) in a case of rabies. Singapore Med J 2000, 41:83-5.

74. Wang SF, Yang CY, Tseng TC, Chen HY. Molecular-Biological Analysis of the First Imported Rabies Case in Taiwan. Epidemiol Bull. 2002, 18:245-55.

75. Hsu YH, Cho LC, Wang LS, Chen LK, Lee JJ, Yang HH. Acute respiratory distress syndrome associated with rabies: a case report. Kaohsiung J Med Sci. 2006; 22: 94-8.

76. ProMED-mail. Published Date: 2012-07-26 18:17:44. Rabies - Taiwan (ex China): canine, human. Archive Number: 20120726.1216441.

77. Kietdumrongwong P, Hemachudha T. Pneumomediastinum as initial presentation of paralytic rabies: a case report. BMC Infect Dis. 2005;5:92

78. ProMED-mail. Published Date: 2009-02-20 19:00:48. Rabies, human - UAE ex India. Archive Number: 20090220.0723.

79. ProMED-mail. Published Date: 2012-09-21 17:15:57. Rabies - China: (ex India) human, exposure unknown. Archive Number: 20120921.1304335

80. Grattan-Smith PJ, O'Regan WJ, Ellis PS, O'Flaherty SJ, McIntyre PB, Barnes CJ. Rabies. A second Australian case, with a long incubation period. Med J Aust. 1992; 156:651-4.

81. McCall KA, Gould AR, Selleck PW, Hooper FT, Westburry HA, Smith JS. Polymerase chain reaction and other laboratory techniques in the diagnosis of long incubation rabies in Australia. Austral Vet J. 1993; 70:84-9.

82. Johnson N, Fooks A, McColl K. Human rabies case with long incubation, Australia. Emerg Infect Dis 2008; 14:1950-1.
